# Puffing of Turmeric (*Curcuma longa* L.) Enhances its Anti-Inflammatory Effects by Upregulating Macrophage Oxidative Phosphorylation

**DOI:** 10.3390/antiox9100931

**Published:** 2020-09-29

**Authors:** Hyunsung Kim, Insu Ban, Yohan Choi, Seungmin Yu, So Jung Youn, Moo-Yeol Baik, Hyungjae Lee, Wooki Kim

**Affiliations:** 1 Department of Food Science and Biotechnology, Graduate School of Biotechnology, Kyung Hee University, Yongin 17104, Korea; lifescienkhs@naver.com (H.K.); qksdlstn@naver.com (I.B.); yo3247@naver.com (Y.C.); dbtmdals1004@khu.ac.kr (S.Y.); mooyeol@khu.ac.kr (M.-Y.B.); 2 Department of Food Engineering, Dankook University, Cheonan 31116, Korea; govl1990@naver.com

**Keywords:** puffing, turmeric, obesity, macrophages, inflammation, oxygen consumption rate

## Abstract

Turmeric (*Curcuma longa* L.), a widely used spice, has anti-inflammatory properties and other health benefits, but the detailed mechanisms of these effects are still poorly understood. Recent advances in assessment of cellular energy metabolism have revealed that macrophage mitochondrial respiration is critical in inflammatory responses. In an effort to enhance the anti-inflammatory function of turmeric with a simple processing method, extract of puffed turmeric was investigated for effect on macrophage energy metabolism. The high-performance liquid chromatography analysis revealed that puffing of turmeric significantly induced the degradation of curcumin to smaller active compounds including vanillic acid, vanillin and 4-vinylguaiacol. The in vitro consumption of oxygen as expressed by the oxygen consumption rate (OCR) was significantly downregulated following lipopolysaccharides stimulation in RAW 264.7 macrophages. Puffed turmeric extract, but not the non-puffed control, reversed the LPS-induced decrease in OCR, resulting in downregulated transcription of the pro-inflammatory genes cyclooxygenase-2 and inducible nitric oxide synthase. Dietary intervention in high-fat diet-induced obese mice revealed that both control and puffed turmeric have anti-obesity effects in vivo, but only puffed turmeric exhibited reciprocal downregulation of the inflammatory marker cluster of differentiation (CD)11c and upregulation of the anti-inflammatory marker CD206 in bone marrow-derived macrophages. Puffed turmeric extract further modulated the low-density lipoprotein/high-density lipoprotein cholesterol ratio toward that of the normal diet group, indicating that puffing is a simple, advantageous processing method for turmeric as an anti-inflammatory food ingredient.

## 1. Introduction

The etiology of inflammation is ambiguous, but many different immune cells are involved and macrophages have been shown to play a key role [1,2]. Macrophages recognize bacterial molecular epitopes, termed pathogen-associated molecular patterns (PAMPs), through plasma membrane-anchored receptors such as toll-like receptors (TLRs) and nucleotide-binding oligomerization domain-like receptors (NLRs). In particular, lipopolysaccharides (LPS), components of Gram-negative bacterial outer membranes, evoke inflammatory responses by binding to TLR4 on macrophage cellular surfaces. A well-defined signaling of LPS-induced macrophage inflammatory response involves a myeloid differentiation primary response 88 and nuclrear factor-κB cascade, resulting in the transcription of signature genes, including inducible nitric oxide synthase (iNOS) and cyclooxygenase (COX)-2. For the molecular pathways recognized, the LPS-induced macrophage inflammatory response model is widely used for in vitro assessment of the anti-inflammatory effects of food ingredients, cosmetics and drugs.

Nobel laureate Dr. Warburg proved that “urgent cells,” including tumor cells, utilize glycolysis and lactate fermentation even with oxygen excess in the cellular microenvironment, a phenomenon termed the Warburg effect [3]. Of interest, it was recently discovered that “urgent cells” include immune cells in an inflammatory state, and LPS-induced macrophages were experimentally determined to switch on lactate fermentation as assessed by decreased oxygen consumption rate (OCR) [4]. Furthermore, the reprogramming of macrophage energy metabolism toward the Krebs cycle, which in turn increases OCR by oxidative phosphorylation, reverses their inflammatory status [5]. In this context, dietary approaches to modulate the Warburg effect for the amelioration of chronic diseases have been proposed [6,7].

The spice turmeric (*Curcuma longa* L.) has been reported to possess multiple health-promoting effects, including anti-inflammatory properties [8,9]. Of interest, it was recently demonstrated that curcumin, a major active compound in turmeric, downregulates the Warburg effect in cancer cells [7]. However, it should also be highlighted that curcumin, due to its hydrophobic structure, is insoluble in water, resulting in the low bioavailability and efficacy [10]. In an effort to enhance its beneficial effects with a simple processing method, it was previously demonstrated that puffing significantly increases turmeric’s antioxidant and anti-inflammatory properties [11], yet the detailed mechanisms remain uncovered. Therefore, the current study investigated the chemical changes of curcuminoids in the turmeric during a puffing process and the effect of puffed turmeric on macrophage energy metabolism using a state-of-the-art technique to quantify cellular consumption of oxygen in a micromilieu. In addition, in an effort to translate the beneficial effect of puffed turmeric in a dietary intervention, a high-fat diet-induced low-grade chronic inflammatory mouse model was adopted in the current study. Here, we report that puffing of turmeric contributes to the degradation of curcumin to more bioavailable active components, resulting in enhancement of macrophage oxidative phosphorylation and the suppression of inflammatory status both in vitro and in vivo.

## 2. Materials and Methods 

### 2.1. Puffing and Extraction of Turmeric

Dried turmeric slices, cultivated in Jindo-gun, Korea, were purchased from Bibong Herb Co. (Yangju-si, Korea). Turmeric was puffed at a pressure of 980 kPa using a customized gun puffing machine in the presence of 4× weighed dried rice for the prevention of carbonization, as previously reported [11,12]. Food grade 70% ethanol purchased from Ethanol Supplies World Co. (Jeonju-si, Korea) was used for puffed turmeric extraction (PTE) as well as non-puffed control turmeric extraction (TE). In brief, 5 g of the ground turmeric was added to 200 mL of 70% ethanol. Subsequently, the mixture was stirred with a magnet at room temperature for 30 min and the vacuum filtrate was stored at −20 °C for subsequent investigations.

### 2.2. Quantitative Analysis of Active Compounds in the Extracts

High-performance liquid chromatography (HPLC) was performed to quantify major curcuminoids, including curcumin (CUR), demethoxycurcumin (DMC) and bisdemethoxycurcumin (BDMC), as well as their degradation products such as ferulic acid (FA), 4-vinylguaiacol (4VG), vanillin (VN) and vanillic acid (VA) in extracts. Briefly, extracts were filtrated using a Millipore filter (pore size 0.45 μm) prior to HPLC analysis. The HPLC system (Agilent 1260 Infinity II, Agilent Technologies, Santa Clara, CA, USA) was equipped with Zorbax SB-C18 column (4.6 × 250 mm, 5 μm) at a flow rate of 1.0 mL/min. The mobile phase consisted of distilled water containing 0.4% acetic acid (A) and acetonitrile (B) in the gradient system using the following conditions: 0–30 min, 8–91% B; 30–39 min, 91–100% B; 39–44 min, 100% B; 44–47 min, 100-8% B; 47-50 min, 8% B. Sample detection was acquired at 260 nm and the injection volume of samples was 10 μL. The seven reference compounds of the curcuminoids and their degradation products with above 98% purity or suitable for HPLC analysis were purchased from Sigma-Aldrich Co. (St. Louis, MO, USA). All the compounds in the samples were identified by comparison of their retention time and spectra with the reference compounds. Quantification was performed using calibration curves of each reference substance.

### 2.3. RAW 264.7 Macrophage Culture

Murine macrophage RAW 264.7 cells were purchased from Korean Cell Line Bank (Seoul, Korea). The cells were cultured on 24-well plates at the density of 1 × 10^5^ cells/mL in Dulbecco’s Modified Eagle’s Medium (DMEM; Welgene, Gyeongsan, Korea) supplemented with 10% fetal bovine serum (FBS; Welgene) and 1% antibiotic/antimycotic solution (10,000 U/mL penicillin G, 10,000 μg/mL streptomycin, and 25 μg/mL amphotericin B; Welgene) at 37 °C in a 5% CO_2_ incubator (Model BB15; Thermo Scientific, Waltham, MA, USA) with either TE, PTE or medium for 24 h. Thereafter, to induce RAW 264.7 cells into an inflammatory status, macrophages were stimulated by LPS (Sigma-Aldrich Co.) at 500 ng/mL for 12 h. Subsequently, the culture media were collected by centrifugation at 300× *g* for 5 min and cell pellets were deep-frozen (SmartCryo^®^ SWUF Ultra-Low-Temperature Freezer; DAIHAN Scientific, Wonju, Korea) at −80 °C for further analyses.

### 2.4. Mitochondrial Oxidative Phosphorylation Assessment

RAW 264.7 cells were aliquoted at 1 × 10^4^ cells/well in Seahorse™ XFp assay plates (Agilent Technologies) followed by treatment with either TE, PTE or medium for 24 h at 37 °C in a 5% CO_2_ incubator. Following extract intervention, LPS (500 ng/mL) was added to the cells for stimulation for 12 h. The stimulated RAW 264.7 cells were further incubated at 37 °C in a non-CO_2_ chamber for 1 h in the presence of DMEM with D-glucose (4500 mg/L), L-glutamine and sodium pyruvate at pH 7.4. The mitochondrial oxidative phosphorylation complex inhibitors oligomycin, carbonyl cyanide 4-(trifluoromethoxy)phenylhydrazone(FCCP) and rotenone/antimycin A (Agilent Technologies) were sequentially added to the cells as previously reported [5]. The oxygen consumption rate (OCR), the indicator of oxidative phosphorylation in mitochondria, was automatically measured by a Seahorse™ XFp analyzer and numerically quantified by dedicated Seahorse^TM^ Wave software (Agilent Technologies) for the assessment of OCR for basal respiration, maximal respiration and ATP production by the following equations:

(1) Basal respiration = OCR before oligomycin—OCR after rotenone/antimycin A
(2) Maximal respiration = Maximum OCR after FCCP—OCR after rotenone/antimycin A
(3) ATP production = OCR before oligomycin—OCR after oligomycin

### 2.5. qRT-PCR Quantification of Pro-Inflammatory mRNA

Total RNA was isolated from harvested RAW 264.7 cells using an MG Total RNA Extraction Kit (MGmed, Seoul, Korea) following the manufacturer’s instructions. The concentration and purity of RNA extract were determined by a spectrophotometer (Nanodrop 2000, Thermo Scientific). The relative quantification for transcription of the pro-inflammatory genes *interleukin* (*IL)-6, tumor necrosis factor (TNF)-α, COX-2,* and *iNOS* was measured by quantitative reverse-transcriptase polymerization chain reaction (qRT-PCR) using the primers shown in Table 1 in the presence of an MG One-step RT-PCR MasterMix (SYBR Green) (MGmed). CFX Connect^TM^ Real-Time System (Bio-Rad, Hercules, CA, USA) was set at the following conditions for amplification of the mRNA: 42 °C for 10 min, 95 °C for 10 min, followed by 39 amplification cycles at 95 °C for 5 s and 55 °C for 30 s. Subsequently, the relative mRNA transcription of target genes was calculated by using 2^−ΔCt^ method for which *glyceraldehyde 3-phosphate dehydrogenase (GAPDH)* served as the internal control gene with Bio-Rad CFX manager software. Specifically, ΔCt was calculated as Ct_target gene_−Ct_GAPDH_, where Ct represents the amplification detection cycle number of an arbitrary threshold.

### 2.6. Animal Study

To investigate the modulation of obesity-induced disorders by either puffed or non-puffed control turmeric, mice were fed a high-fat diet (HFD) in accordance with guidelines approved by the Kyung Hee University Institutional Animal Care and Use Committee (approval number KHGASP-19-135). Briefly, 40 male C57BL/6 mice at 4 weeks of age were purchased from Raon Bio (Yongin, Korea). The mice were allocated to 4 groups (*n* = 10) and fed a rodent chow diet for 1 week for acclimation. Each experimental diet was modified from the AIN-76A semi-purified diet, and the HFD contained 45% kcal fat from corn oil and lard (Table 2). Ground powder of either non-puffed control turmeric (HFD + T) or puffed turmeric (HFD + PT) replaced the dietary fiber cellulose. After the adaptation period, mice were fed with respective diets for 12 weeks ad libitum under a climate-controlled 12:12 h light:dark cycle, and body weights of individual mice were recorded weekly. Following the dietary intervention, the mice were euthanized by CO_2_ inhalation and the blood was collected by a cardiac puncture. Subsequently, the blood glucose levels were quantified using an Accu-Chek^®^ Performa Kit (Roche Diabetes Care, Mannheim, Germany). Tibias and femurs of the mice were collected for the isolation of bone marrow cells in an aseptic environment, followed by staining with anti-CD11c-R-phycoerythrin (eBiosciences, San Diego, CA, USA) and anti-CD206-fluorescein isothiocyanate (eBiosciences) monoclonal antibodies and flow cytometric analysis using Accuri^TM^ C6 (BD Biosciences, Franklin Lakes, NJ, USA). Collected blood was further stored at 4 °C for 3–4 h in order to isolate serum by centrifugation at 300× *g* for 5 min. Serum insulin was assessed by using the Ultra-Sensitive Mouse Insulin enzyme-linked immunosorbent assay (ELISA) Kit (Crystal Chem Inc., Chicago, IL, USA) and total, high-density lipoprotein (HDL) and low-density lipoprotein (LDL) cholesterol were analyzed with the Cholesterol Assay Kit—HDL and LDL/very low-density lipoprotein (VLDL) (Abcam, Cambridge, MA, USA), according to the manufacturer’s instructions.

### 2.7. Statistical Analysis

Data are representative of repeated experiments and are presented as mean ± standard error of the mean (SEM). Statistical significance at *p* < 0.05 was determined by one-way analysis of variance (ANOVA) followed by Tukey’s post-hoc multiple comparisons test using GraphPad Prism 8 software (La Jolla, CA, USA).

## 3. Results

### 3.1. Degradation of Curcuminoids in Turmeric by Puffing

**Figure 1:**
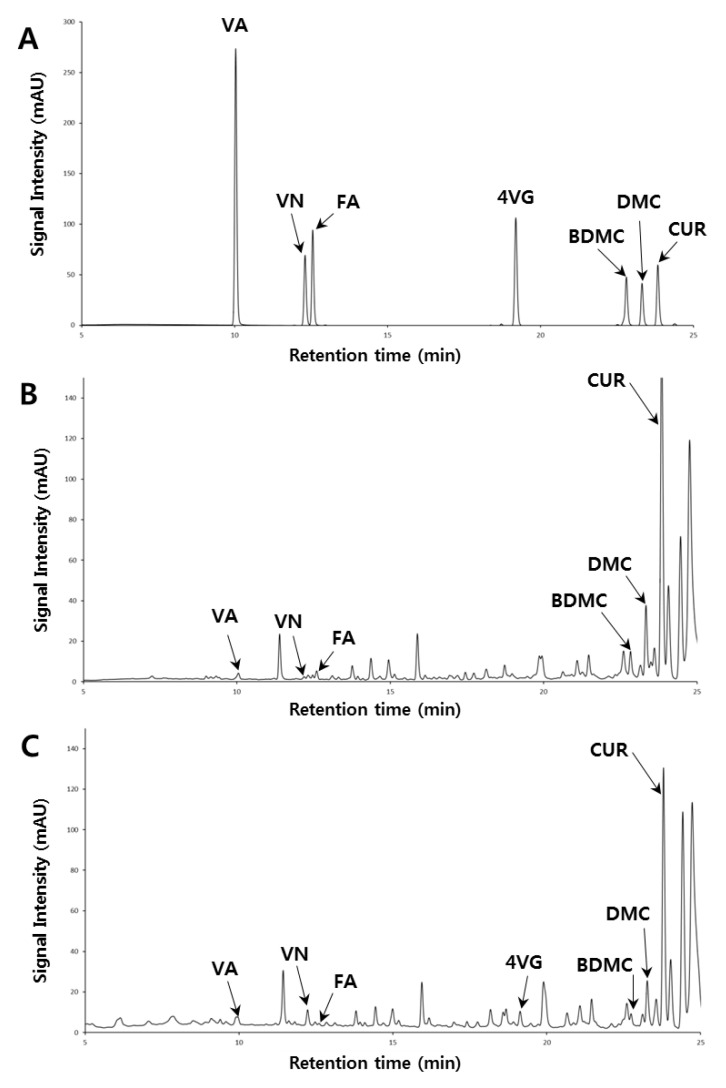
Representative high-performance liquid chromatograms of (**A**) seven reference compounds including curcuminoids and their degradation products, (**B**) turmeric extract, TE, and (**C**) puffed turmeric extract, PTE for the quantitative analysis. VA, vanillic acid; VN, vanillin; FA, ferulic acid; 4VG, 4-vinylguaiacol; BDMC, bisdemethoxycurcumin; DMC, demethoxycurcumin; CUR, curcumin.

The HPLC analysis (Figure 1) of TE revealed that CUR and its analogues BDMC and DMC are the major active compounds of non-puffed control turmeric (Table 3). In contrast, puffing process significantly decreased the quantity of those curcuminoids in the extracts, while their degradation compounds VA, VN and 4VG were drastically increased (Figure 1).

### 3.2. Puffing of Turmeric Enhances Mitochondrial Respiration in Macrophages

**Figure 2:**
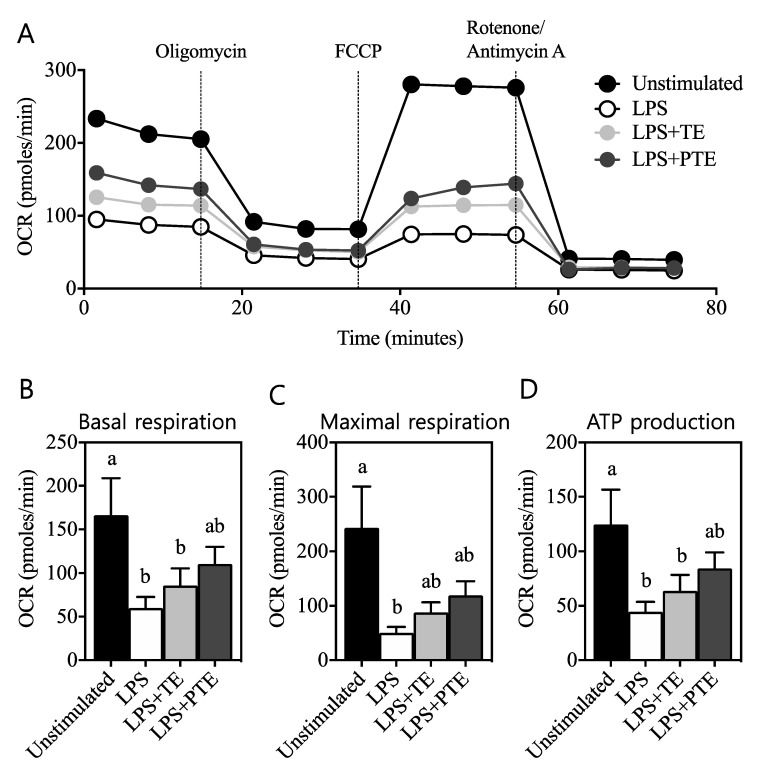
Mitochondrial respiration as assessed by oxygen consumption rate (OCR) of RAW 264.7 cells was quantified in a real-time manner by an extracellular flux analyzer (**A**). OCR for basal respiration (**B**), maximal respiration (**C**), and ATP production (**D**) was further judged by treatment of cells with specific inhibitors or uncouplers, as described in the Materials and Methods. Different letters indicate that the means are significantly different at *p* < 0.05 within the panel.

The cellular oxygen consumption rate (OCR), which represents mitochondrial respiration, was sensitively measured in a 96-well plate in real time by an extracellular flux analyzer, as shown in Figure 2. At a basal level prior to treatment of any cellular modulator, OCR of unstimulated cells exhibited the highest values, over 200 pmoles/min (Figure 2A, black circles). LPS treatment of the cells dramatically reduced OCR to around 100 pmoles/min (Figure 2A, white circles), which was reversed by the addition of TE (Figure 2A, light grey circles). Of interest, the treatment of cells with PTE further recovered the LPS-induced downregulation of OCR toward non-stimulated status (Figure 2A, dark grey circles).

Following the sequential treatments of cells with well-defined inhibitors or uncouplers of mitochondrial functions, OCR for basal respiration, maximal respiration, and adenosine triphosphate (ATP) production were calculated as shown in Figure 2B–D. Unstimulated cells exhibited the highest OCR for basal respiration (166.20 ± 42.71 pmoles/min, Figure 2B, black bar), which was significantly decreased (60.00 ± 12.60 pmoles/min, white bar) by LPS stimulation. TE treatment to LPS-stimulated cells (85.74 ± 19.76 pmoles/min, light grey bar) did not demonstrate any difference to LPS-only activated cells, yet a statistical difference with unstimulated cells was observed (*p* < 0.05). In contrast, PTE-treated cells in the presence of LPS presented OCR for basal respiration at a value of 110.63 ± 19.77 pmoles/min (dark grey bar), without any statistical significance to both unstimulated cells and LPS-only-treated cells (*p* > 0.05). OCR analysis for maximal respiration (Figure 2C) revealed that unstimulated macrophages required the most oxygen molecules at 242.0 ± 75.85 pmoles/min (black bar), and LPS treatment significantly lowered that value to 50.24 ± 11.05 pmoles/min (white bar) (*p* < 0.05). Maximal respiration OCR following treatment by either TE (87.68 ± 18.77 pmoles/min, light grey bar) or PTE (119.2 ± 26.07 pmoles/min, dark grey bar) tended to reverse the LPS-induced reduction of OCR without any statistical power to both LPS-only-treated and unstimulated cells. The cellular oxygen requirement for ATP production of unstimulated macrophages dropped from 124.6 ± 32.03 pmoles/min (Figure 2D, black bar) for unstimulated cells to 44.39 ± 9.21 pmoles/min for LPS-treated cells (white bar). Following TE intervention to the cells, OCR required for ATP production (63.80 pmoles/min for LPS + TE, light grey bar) exhibited a comparable rate to the LPS-only-treated group, yet a significant difference to unstimulated cells was retained. Following PTE treatment (84.49 ± 14.54 pmoles/min, dark grey bar), however, ATP production OCR exhibited an identical pattern to that of basal respiration, indicating the differential modulation of cellular energy metabolism by TE vs. PTE.

### 3.3. Suppressed Transcription of Pro-Inflammatory mRNA by PTE

**Figure 3:**
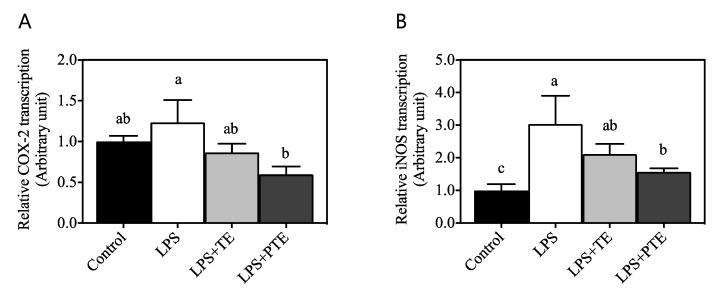
Transcription assessment of pro-inflammatory genes cyclooxygenase (COX)-2 (**A**) and inducible nitric oxide synthase (iNOS) (**B**) by quantitative reverse-transcriptase polymerization chain reaction. Relative amount of mRNA was normalized to non-stimulated control cells. Different letters indicate that the means are significantly different at *p* < 0.05 within the panel.

LPS-stimulated macrophages have been reported to induce transcription of the signature pro-inflammatory genes COX-2 and iNOS [16], which further mediate the production of prostaglandin E_2_ and nitric oxide, respectively. In order to determine if OCR upregulated by PTE further modulates inflammatory responses, transcription of these genes was assessed by qRT-PCR. As shown in Figure 3A, transcription of COX-2 was determined by the relative transcription to non-stimulated control cells (1.00 ± 0.07 arbitrary unit). Following LPS stimulation, COX-2 transcription (1.23 ± 0.28) exhibited an increased inclination without any statistical significance to control cells (*p* > 0.05). The addition of non-puffed turmeric extract (LPS + TE) demonstrated no effect on COX-2 transcription (0.87 ± 0.11), whereas puffed turmeric extract (LPS + PTE) significantly downregulated COX-2 transcription (0.60 ± 0.10) compared with LPS-stimulated cells (*p* < 0.05). iNOS transcription (Figure 3B) was also significantly increased by LPS treatment (3.03 ± 0.87) compared with non-stimulated control cells (1.00 ± 0.19, *p* < 0.05). TE treatment to LPS-activated macrophages (2.11 ± 0.31) did not exhibit any statistical difference, but PTE drastically downregulated LPS-induced increase of iNOS transcription (1.57 ± 0.11, *p* < 0.05), confirming the distinct regulation of macrophages by TE vs. PTE.

### 3.4. Inhibition of High-Fat Diet-Induced Weight Gain by TE and PTE

**Figure 4:**
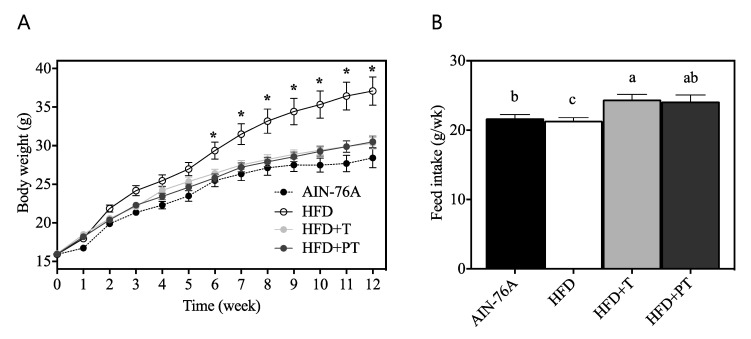
Dietary intervention of turmeric and puffed turmeric in high-fat diet-induced obese mice. Body weight gain (**A**) and feed intake (**B**) were observed. * Significantly different from AIN-76A control diet group at specific week (*p* < 0.05). Different letters indicate significantly different means among diet groups.

To investigate if puffing of turmeric affects low-grade chronic inflammatory status in vivo, obesity in C57BL/6 male mice was induced by a high-fat diet (HFD). In particular, 45% of dietary energy was supplied in the form of fat with or without turmeric (T) or puffed turmeric (PT) as a replacement for dietary fiber cellulose. Throughout the 12 weeks of ad libitum feeding, body weight changes of individual mice were recorded weekly. HFD-fed mice (Figure 4A, open circle) exhibited significant weight gain starting at 6 weeks of feeding as compared with the normal AIN-76A diet-fed control group (closed circle, *p* < 0.05). Of interest, the addition of turmeric as well as puffed turmeric inhibited the HFD-induced body weight gain (light/dark grey circles). There was no statistical significance between HFD + T vs. AIN-76A, HFD + PT vs. AIN-76A and HFD + T vs. HFD + PT throughout the dietary intervention. The food intake of mice was also investigated because body weight change can be affected not only by caloric density but also the feeding load. Throughout the feeding period, the AIN-76A diet was consumed at 21.71 ± 0.54 g-diet/week (Figure 4B). Mice consumed a slightly but significantly less amount of HFD (21.35 ± 0.44 g-diet/week (*p* < 0.05), an energy-dense diet. Consumption of HFD + T and HFD + PT was determined to be 24.39 ± 0.77 g-diet/week and 24.12 ± 0.95 g-diet/week, respectively.

### 3.5. Reciprocal Regulation of Pro- and Anti-Inflammatory Markers on Bone Marrow-Derived Macrophages (BMDM) by PT

**Figure 5:**
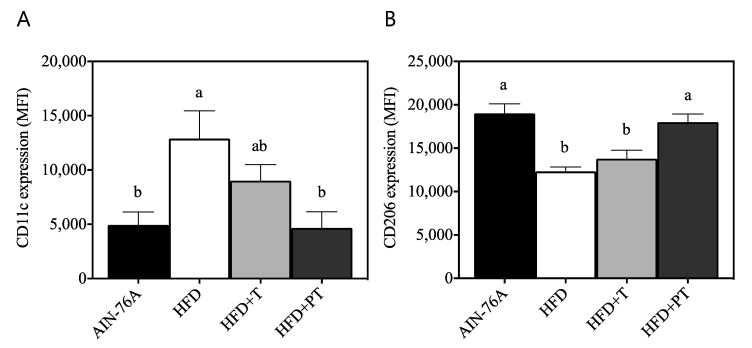
Surface expression of activation markers on bone marrow-derived macrophages as determined by fluorescent antibody staining and flow cytometric analysis. Pro-inflammatory marker cluster of differentiation (CD)11c (**A**) and anti-inflammatory marker CD206 (**B**) were reciprocally modulated by HFD and PT. Different letters indicate significantly different means among diet groups within the panel.

Following observation of anti-obesity effects of both T and PT, anti-inflammatory markers on bone marrow-derived macrophages (BMDM) were quantified by specific staining of fluorescence conjugated mAbs followed by flow cytometric analysis, as expressed by mean fluorescence intensity (MFI, arbitrary unit). The expression of CD11c, a well-known macrophage surface marker for pro-inflammatory M1 subsets [17], was significantly (*p* < 0.05) increased in the HFD group (Figure 5A, 12,898 ± 2558) vs. the AIN-76A diet control (4929 ± 1212). The addition of turmeric (HFD + T, 9011 ± 1501) did not exhibit a significant difference to HFD only (*p* > 0.05), where puffed turmeric (HFD + PT, 4656 ± 1498) significantly suppressed surface display of CD11c comparable to the AIN-76A diet group. Expression of the anti-inflammatory marker CD206 was further assessed, and a significant decrease in the HFD group (12,327 ± 517 vs. AIN-76A 18,992 ± 1122, *p* < 0.05) was observed (Figure 5B). In accordance to CD11c expression, the turmeric diet (HFD + T, 13,791 ± 974) did not affect HFD-induced alteration of CD206 expression, but puffed turmeric exhibited a notable impact on CD206 expression (HFD + PT, 18,006 ± 926), comparable to the AIN-76A diet group. 

### 3.6. Effects of PT Diet on Obesity-Induced Dyslipidemia

**Figure 6:**
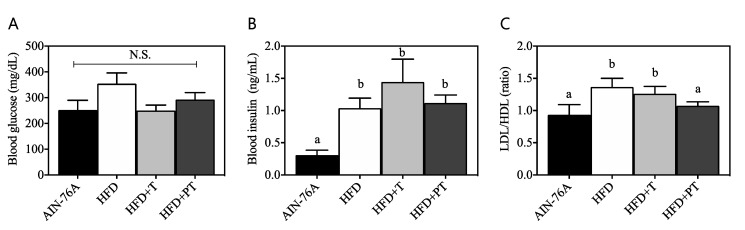
Measurement of serum glucose (**A**), insulin (**B**) and low-density lipoprotein (LDL)/(high-density lipoprotein) HDL ratio (**C**). Statistical significances within the panel are expressed with the letters (*p* < 0.05). N.S. indicates no significance between the dietary interventions.

Following dietary intervention, serum markers including fasting glucose, insulin and LDL/HDL cholesterol were quantified in order to assess the effects of diet on chronic states. As seen in Figure 6A, diets did not statistically affect fasting glucose in serum, even though a trend in increased glucose was observed in the HFD group (354.5 ± 41.5 mg/dash (d)L) vs. the AIN-76A group (252 ± 37.8 mg/dL). Serum glucose from HFD + T and HFD + PT mice was determined to be 250 ± 21.4 mg/dL and 292.5 ± 27.1 mg/dL, respectively. On the other hand, serum insulin (Figure 6B) was significantly increased by feeding of HFD (1.04 ± 0.16 ng/mL) vs. AIN-76A (0.31 ± 0.08 ng/mL), for which the addition of turmeric (HFD + T, 1.44 ± 0.35 ng/mL) and puffed turmeric (HFD + PT, 1.12 ± 0.12 ng/mL) exhibited no effect. The ratio of LDL/HDL cholesterol, a well-defined hyperlipidemia marker for obesity-related chronic diseases [18], was subsequently determined in sera. Serum from AIN-76A mice exhibited an LDL/HDL ratio of 0.93 ± 0.16 (Figure 6C, black), which was significantly increased by HFD to 1.37 ± 0.14 (white). Following the dietary intervention with puffed turmeric, however, the LDL/HDL ratio was decreased to be 1.07 ± 0.06 (dark grey), comparable to the AIN-76A group, but non-puffed turmeric (HFD + T, 1.26 ± 0.11) did not affect the HFD-induced increase.

## 4. Discussion

Turmeric has long been used as a spice as well as a medicinal herb in Asian countries [19]. The major active compounds of turmeric are curcuminoids, consisting of CUR, DMC and BDMC [20]. Many studies have shown the beneficial health effects of these chemicals, but bioavailability remains a concern due to their low solubility in water [21]. In order to enhance their water solubility and subsequent bioavailability, various approaches including nano-particlization [22], encapsulation [23] and bioconversion [24] have been introduced. It was also reported that emulsion formation of curcumin with oils aid in the bioaccessibility through the lipid digestion and absorption system in the gastrointestinal tract [25]. In an extension to those studies, we previously reported that puffing, a simple processing method, successfully enhanced turmeric’s antioxidant and anti-inflammatory effects in vitro, although the detailed mechanisms are still unknown [11]. Puffing is a physical transformation that increases porosity and causes chemical reactions, including the well-defined Maillard reaction, the products of which have antioxidant capacities [26]. The current study further demonstrated that the high heat and pressure during puffing induced degradation of curcuminoids to smaller compounds including VA, VN and 4VG (Figure 1 and Table 3). In this study, a new HPLC method has been developed to quantify seven major compounds of TE and PTE by an injection on HPLC since no simultaneous detection of the three curcuminoids and their four degradation products has been reported, even though previous studies have performed HPLC analyses of curcuminoids and volatile compounds [27] or degradation compounds from roasted turmeric [28] at once. In fact, studies have revealed that those degradation products have higher bioavailability and antioxidant capacities as compared to the parental curcuminoids [29,30]. Similarly, puffing also increased the antioxidant properties of coffee beans by increment of phenolic compounds [31,32], indicating that it is a simple and promising method for functional development of plant-derived food materials. 

It is well-known that eukaryotic cells make pyruvate through glycolysis, which is then oxidized to acetyl-coenzyme A in aerobic conditions. The acetyl- coenzyme A is further oxidized through the tricarboxylic acid (TCA) cycle and produces nicotinamide adenine dinucleotide (NADH) and flavin adenine dinucleotide (FADH_2_). In mitochondrial membranes, a proton gradient produces ATP by consumption of oxygen, a process called mitochondrial respiration. In anaerobic conditions, the cells cannot conduct mitochondrial respiration due to insufficient oxygen. Interestingly, however, even when oxygen is abundant, specific cells, including cancer and inflammatory cells, utilize glycolysis and lactate fermentation, but not mitochondrial respiration, a process termed the Warburg effect [3,6,33]. 

Macrophages play a critical function in innate immunity by cueing the inflammatory signals [34]. Murine cell line RAW 264.7 macrophages are widely utilized in the screening of anti-inflammatory properties of natural products. Following LPS engagement with TLR4 on the macrophage surface, intracellular signaling occurs via the MyD88–NFκB axis. These signaling pathways initiate transcription of inflammatory genes including IL-6, TNF-α, COX-2 and iNOS. Upon classically activated stimulation of “naïve” macrophages, polarized pro-inflammatory macrophages are prone to utilize glycolysis for faster production of ATP, while reserving fatty acid synthesis for the production of lipid mediators for inflammatory cascades [35]. In fact, recent studies indicate that the energy metabolism in inflammatory macrophages is complicated, and they have potential as a target for dietary amelioration of inflammation [36].

Therefore, in the current study, it was hypothesized that puffed turmeric and its extract increase macrophage mitochondrial respiration for regulation of inflammatory responses. In an in vitro inflammatory model of RAW 264.7 cells stimulated with LPS, mitochondrial respiration, as assessed by cellular OCR, was significantly decreased, confirming the Warburg effect in the model cells (Figure 2A). Of interest, the addition of non-puffed control turmeric extract (TE) recovered the LPS-induced OCR decrease, and puffed turmeric extract (PTE) exhibited an even stronger effect (Figure 2A). The detailed OCRs for basal respiration, maximal respiration capacity and ATP production were dissected by treatment of oligomycin, FCCP and rotenone/antimycin A, respectively [37]. As shown in Figure 2B, those specific OCRs were downregulated by LPS treatment, and PTE, but not TE, recovered the cells from the LPS-induced inflammatory status. Subsequently, the expression of the signature pro-inflammatory genes COX-2 and iNOS was quantified by qRT-PCR. As compared to LPS-induced upregulation of transcription of both genes, PTE but not TE suppressed the transcription of those genes (Figure 3), confirming that increased OCR affects the suppressed inflammatory responses in macrophages. Together with the previous observations of reduced cytokine secretion (IL-6 and TNF-α) and surface marker expression (CD80 and CD86) [11], the current study clearly demonstrates that puffing increases the anti-inflammatory effects of turmeric.

In an extension to our in vitro observations of enhanced anti-inflammatory effects of turmeric by puffing, dietary in vivo functions were further investigated in HFD-induced obese mice. Following 12-week feeding of HFD (45% kcal as fat), body weight of the mice drastically increased compared with AIN-76A control mice (Figure 4A). Interestingly, replacement of dietary fiber cellulose (50 g/kg diet) with either non-puffed turmeric (T) or puffed turmeric (PT) inhibited HFD-induced weight gain, resulting in a similar body weight to AIN-76-fed mice (Figure 4A). The quantification of diet consumption revealed that mice consumed less of the energy-dense HFD than the AIN-76A diet (Figure 4B). In this regard, it was previously reported that HFD-induced obesity causes insulin insensitivity and reduced leptin secretion, resulting in decreased appetite in mice [38]. Following addition of T or PT at the expense of cellulose, food consumption was increased (Figure 4B), indicating that the anti-obese effects of T and PT were not a result of less ingestion. Following observation of reduced weight gain by T and PT, their effects on macrophage functioning was determined by assessment of the surface molecules CD11c and CD206 on bone marrow-derived macrophages. CD11c was previously reported to be a marker of pro-inflammatory M1 macrophages, whereas CD206 is used for the detection of anti-inflammatory M2 macrophages [39]. The puffed turmeric reciprocally regulated those markers by reducing CD11c and increasing CD206 as compared to the HFD group (Figure 5). In comparison, consumption of non-puffed turmeric exhibited a marginal but nonsignificant effect, confirming the differential regulation of inflammation by non-puffed vs. puffed turmeric. The etiology of inflammation is very complicated, and it was previously reported that the mechanisms of dyslipidemia and inflammation are closely relevant [40,41]. Therefore, we further investigated if PT affects HFD-induced dyslipidemia by assessment of blood glucose, insulin and cholesterols. Glucose and insulin in fasting blood were not altered by dietary treatment of either T or PT in the HFD condition (Figure 6A,B). However, the LDL/HDL ratio, which was increased by the HFD, was significantly downregulated by PT but not T (Figure 6C).

Overall, the current study clearly demonstrates that puffing is a simple and promising processing method of turmeric for its application in functional foods for prevention and/or amelioration of inflammation. However, beyond the curcuminoid degradation, more studies are required to discover the products generated by puffing of turmeric. It was previously reported that Maillard reaction products, which should be generated by puffing, aid in ameliorating inflammation in mice [42], emphasizing the need for detailed studies demonstrating these molecular mechanisms.

## Figures and Tables

**Table 1:** Primer sequences for quantitative real-time polymerase chain reaction quantification of pro-inflammatory mRNA.

mRNA	Sequence (5′ → 3′)		References
interleukin-6	Forward	GTACTCCAGAAGACCAGAGG	[13]
	Reverse	TGCTGGTGACAACCACGGCC	
tumor necrosis factor- α	Forward	CCTGTAGCCCACGTCGTAGC	[13]
	Reverse	CTCAGCACCCACCCGCTCA	
cyclooxygenase-2	Forward	TCTCAGCACCCACCCGCTCA	[14]
	Reverse	TTTGACCTCAGCGCTGAGTTG	
inducible nitric oxide synthase	Forward	CCCTTCCGAAGTTTCTGGCAGCAGC	[15]
	Reverse	GGCTGTCAGAGCCTCGTGGCTTTGG	
glyceraldehyde-3-phosphate dehydrogenase	Forward	CACTCACGGCAAATTCAACGGC	[11]
	Reverse	CCTTGGCAGCACCAGTGGATGCAGG	

**Table 2:** Experimental diet composition.

Ingredients	Composition (g/kg)			
	AIN-76A ^(1)^	HFD	HFD + T	HFD + PT
Casein	200	200	200	200
DL-methionine	3	3	3	3
Corn starch	150	111	111	111
Sucrose	500	370	370	370
Corn oil	50	30	30	30
Lard	-	170	170	170
Mineral mix S10001	35	35	35	35
Vitamin mix V10001	10	10	10	10
Choline bitartarate	2	2	2	2
Cellulose	50	50	-	-
Turmeric	-	-	50	-
Puffed turmeric	-	-	-	50

^(1)^ Experimental diets were modified from the AIN-76A diet. The cellulose in the HFD was replaced by turmeric or puffed turmeric in HFD + T or HFD + PT respectively, for an isocaloric equivalence.

**Table 3:** Chemical changes in turmeric by puffing (μg/g dried turmeric).

	VA ^(1)^	VN	FA	4VG	BDMC	DMC	CUR
TE	6.00 ± 1.30	21.49 ± 0.63	23.34 ± 0.19	N/D ^(2)^	23.13 ± 0.61	91.66 ± 2.14	397.42 ± 3.75
PTE	9.87 ± 0.55 *	90.65 ± 3.83 *	5.67 ± 0.80 *	99.57 ± 8.77 *	10.07 ± 0.87 *	60.86 ± 5.83 *	300.57 ± 26.06 *

^(1)^ VA, vanillic acid; FA, ferulic acid; VN, vanillin; 4VG, 4-vinylguaiacol; BDMC, bisdemethoxycurcumin; DMC, demethoxycurcumin; CUR, curcumin. ^(2)^ N/D, not detected. * Significantly different from TE at *p* < 0.05 (*n* = 3)

## References

[1] Koh T.J., DiPietro L.A. (2011). Inflammation and wound healing: The role of the macrophage. Expert Rev. Mol. Med..

[2] Kim L., Kim J.Y. (2019). Chondroprotective effect of curcumin and lecithin complex in human chondrocytes stimulated by IL-1β via an anti-inflammatory mechanism. Food Sci. Biotechnol..

[3] Liberti M.V., Locasale J.W. (2016). The Warburg effect: How does it benefit cancer cells?. Trends Biochem. Sci..

[4] Mills E.L., O’Neill L.A. (2016). Reprogramming mitochondrial metabolism in macrophages as an anti-inflammatory signal. Eur. J. Immunol..

[5] Yu S., Go G.W., Kim W. (2019). Medium chain triglyceride (MCT) oil affects the immunophenotype via reprogramming of mitochondrial respiration in murine macrophages. Foods.

[6] Zhao Y., Butler E.B., Tan M. (2013). Targeting cellular metabolism to improve cancer therapeutics. Cell Death Dis..

[7] Siddiqui F.A., Prakasam G., Chattopadhyay S., Rehman A.U., Padder R.A., Ansari M.A., Irshad R., Mangalhara K., Bamezai R.N.K., Husain M. (2018). Curcumin decreases Warburg effect in cancer cells by down-regulating pyruvate kinase M2 via mTOR-HIF1α inhibition. Sci. Rep..

[8] Krup V., Prakash L.H., Harini A. (2013). Pharmacological Activities of Turmeric (*Curcuma longa linn*): A Review. J. Homeopath. Ayurvedic Med..

[9] Chainani-Wu N. (2003). Safety and Anti-Inflammatory Activity of Curcumin: A Component of Tumeric (*Curcuma longa*). J. Altern. Complement. Med..

[10] Anand P., Kunnumakkara A.B., Newman R.A., Aggarwal B.B. (2007). Bioavailability of Curcumin: Problems and Promises. Mol. Pharm..

[11] Choi Y., Ban I., Lee H., Baik M.-Y., Kim W. (2019). Puffing as a Novel Process to Enhance the Antioxidant and Anti-Inflammatory Properties of *Curcuma longa* L. (Turmeric). Antioxidants.

[12] Kwon Y., Yu S., Choi G.S., Kim J.H., Baik M., Su S.T., Kim W. (2019). Puffing of Rehmannia glutinosa enhances anti-oxidant capacity and down-regulates IL-6 production in RAW 264.7 cells. Food Sci. Biotechnol..

[13] Yang S.-J., Lee J.-E., Lim S.-M., Kim Y.-J., Lee N.-K., Paik H.-D. (2019). Antioxidant and immune-enhancing effects of probiotic Lactobacillus plantarum 200655 isolated from kimchi. Food Sci. Biotechnol..

[14] Yayeh T., Oh W.J., Park S.-C., Kim T.-H., Cho J.Y., Park H.-J., Lee I.-K., Kim S.-K., Hong S.-B., Yun B.-S. (2012). Phellinus baumii ethyl acetate extract inhibits lipopolysaccharide-induced iNOS, COX-2, and proinflammatory cytokine expression in RAW264.7 cells. J. Nat. Med..

[15] Chiou W.-F., Chen C.-F., Lin J.-J. (2000). Mechanisms of suppression of inducible nitric oxide synthase (iNOS) expression in RAW 264.7 cells by andrographolide. Br. J. Pharmacol..

[16] Lee S.H., Soyoola E., Chanmugam P., Hart S., Sun W., Zhong H., Liou S., Simmons D., Hwang D. (1992). Selective expression of mitogen-inducible cyclooxygenase in macrophages stimulated with lipopolysaccharide. J. Biol. Chem..

[17] Ono Y., Nagai M., Yoshino O., Koga K., Nawaz A., Hatta H., Nishizono H., Izumi G., Nakashima A., Imura J. (2018). CD11c+ M1-like macrophages (MΦs) but not CD206+ M2-like MΦ are involved in folliculogenesis in mice ovary. Sci. Rep..

[18] Assunção M.L., Ferreira H.S., dos Santos A.F., Cabral C.R., Florêncio T.M.M.T. (2009). Effects of Dietary Coconut Oil on the Biochemical and Anthropometric Profiles of Women Presenting Abdominal Obesity. Lipids.

[19] Maheshwari R.K., Singh A.K., Gaddipati J., Srimal R.C. (2006). Multiple biological activities of curcumin: A short review. Life Sci..

[20] Nelson K.M., Dahlin J.L., Bisson J., Graham J., Pauli G.F., Walters M.A. (2017). The Essential Medicinal Chemistry of Curcumin. J. Med. Chem..

[21] Cui J., Yu B., Zhao Y., Zhu W., Li H., Lou H., Zhai G. (2009). Enhancement of oral absorption of curcumin by self-microemulsifying drug delivery systems. Int. J. Pharm..

[22] Tiwari S.K., Agarwal S., Seth B., Yadav A., Nair S., Bhatnagar P., Karmakar M., Kumari M., Chauhan L.K.S., Patel D.K. (2014). Curcumin-Loaded Nanoparticles Potently Induce Adult Neurogenesis and Reverse Cognitive Deficits in Alzheimer’s Disease Model via Canonical Wnt/β-Catenin Pathway. ACS Nano.

[23] Sari T.P., Mann B., Kumar R., Singh R.R.B., Sharma R., Bhardwaj M., Athira S. (2015). Preparation and characterization of nanoemulsion encapsulating curcumin. Food Hydrocoll..

[24] Majeed A., Majeed M., Thajuddin N., Arumugam S., Ali F., Beede K., Adams S.J., Gnanamani M. (2019). Bioconversion of curcumin into calebin-A by the endophytic fungus Ovatospora brasiliensis EPE-10 MTCC 25236 associated with Curcuma caesia. AMB Express.

[25] Shah B.R., Zhang C., Li Y., Li B. (2016). Bioaccessibility and antioxidant activity of curcumin after encapsulated by nano and Pickering emulsion based on chitosan-tripolyphosphate nanoparticles. Food Res. Int..

[26] Wang H.-Y., Qian H., Yao W.-R. (2011). Melanoidins produced by the Maillard reaction: Structure and biological activity. Food Chem..

[27] Chao I.-C., Wang C.-M., Li S.-P., Lin L.-G., Ye W.-C., Zhang Q.-W. (2018). Simultaneous Quantification of Three Curcuminoids and Three Volatile Components of Curcuma longa Using Pressurized Liquid Extraction and High-Performance Liquid Chromatography. Molecules.

[28] Esatbeyoglu T., Ulbrich K., Rehberg C., Rohn S., Rimbach G. (2015). Thermal Stability, Antioxidant, and Anti-inflammatory Activity of Curcumin and its Degradation Product 4-vinyl guaiacol. Food Funct..

[29] Shen L., Liu C.-C., An C.-Y., Ji H.-F. (2016). How does curcumin work with poor bioavailability? Clues from experimental and theoretical studies. Sci. Rep..

[30] Shen L., Ji H.F. (2009). Contribution of degradation products to the anticancer activity of curcumin. Clin. Cancer Res..

[31] Kim W., Kim S.-Y., Kim D.-O., Kim B., Baik M.-Y. (2018). Puffing, a Novel Coffee Bean Processing Technique for the Enhancement of Extract Yield and Antioxidant Capacity. Food Chem..

[32] Han S.H., Ko B.S., Ahn S.H., Noh D.O., Suh H.J. (2017). Comparison of the antioxidant activities of roasted and explosive puffed coffees. Int. J. Food Sci. Technol..

[33] Zhu L., Zhao Q., Yang T., Ding W., Zhao Y. (2015). Cellular metabolism and macrophage functional polarization. Int. Rev. Immunol..

[34] Fujiwara N., Kobayashi K. (2005). Macrophages in Inflammation. Curr. Drug Target Inflamm. Allergy.

[35] Galván-Peña S., O’Neill L.A.J. (2014). Metabolic reprograming in macrophage polarization. Front. Immunol..

[36] Batista-Gonzalez A., Vidal R., Criollo A., Carreño L.J. (2020). New Insights on the Role of Lipid Metabolism in the Metabolic Reprogramming of Macrophages. Front. Immunol..

[37] Iuso A., Repp B., Biagosch C., Terrile C., Prokisch H. (2017). Assessing Mitochondrial Bioenergetics in Isolated Mitochondria from Various Mouse Tissues Using Seahorse XF96 Analyzer. Methods in Molecular Biology.

[38] Moraes J.C., Coope A., Morari J., Cintra D.E., Roman E.A., Pauli J.R., Romanatto T., Carvalheira J.B., Oliveira A.L.R., Saad M.J. (2009). High-Fat Diet Induces Apoptosis of Hypothalamic Neurons. PLoS ONE.

[39] Murray P.J., Allen J.E., Biswas S.K., Fisher E.A., Gilroy D.W., Goerdt S., Gordon S., Hamilton J.A., Ivashkiv L.B., Lawrence T. (2014). Macrophage Activation and Polarization: Nomenclature and Experimental Guidelines. Immunity.

[40] Esteve E., Ricart W., Fernández-Real J.M. (2005). Dyslipidemia and inflammation: An evolutionary conserved mechanism. Clin. Nutr..

[41] Oh I., Baek E.J., Lee D.-H., Choi Y.H., Bae I.Y. (2019). Anti-obesity and anti-inflammatory effects of ginseng vinegar in high-fat diet fed mice. Food Sci. Biotechnol..

[42] Al Amir I., Dubayle D., Héron A., Delayre-Orthez C., Anton P.M. (2017). Maillard reaction products from highly heated food prevent mast cell number increase and inflammation in a mouse model of colitis. Nutr. Res..

